# A Severe Case of Buruli Ulcer Disease with Pleural Effusions

**DOI:** 10.1371/journal.pntd.0002868

**Published:** 2014-06-19

**Authors:** Fred S. Sarfo, William Thompson, Richard O. Phillips, Albert Paintsil, Mohammed K. Abass, Michael Frimpong, Justice Abotsi, Kingsley Asiedu, Mark H. Wansbrough-Jones

**Affiliations:** 1 Komfo Anokye Teaching Hospital, Kumasi, Ghana; 2 School of Medical Sciences, Kwame Nkrumah University of Science and Technology, Kumasi, Ghana; 3 Agogo Presbyterian Hospital, Ministry of Health, Agogo, Ghana; 4 Korle-Bu Teaching Hospital, Accra, Ghana; 5 Kumasi Center for Collaborative Research, Kumasi, Ghana; 6 World Health Organisation, Geneva, Switzerland; 7 St. George's University of London, London, United Kingdom; Swiss Tropical and Public Health Institute, Switzerland

## Presentation of Case

**Figure 1:**
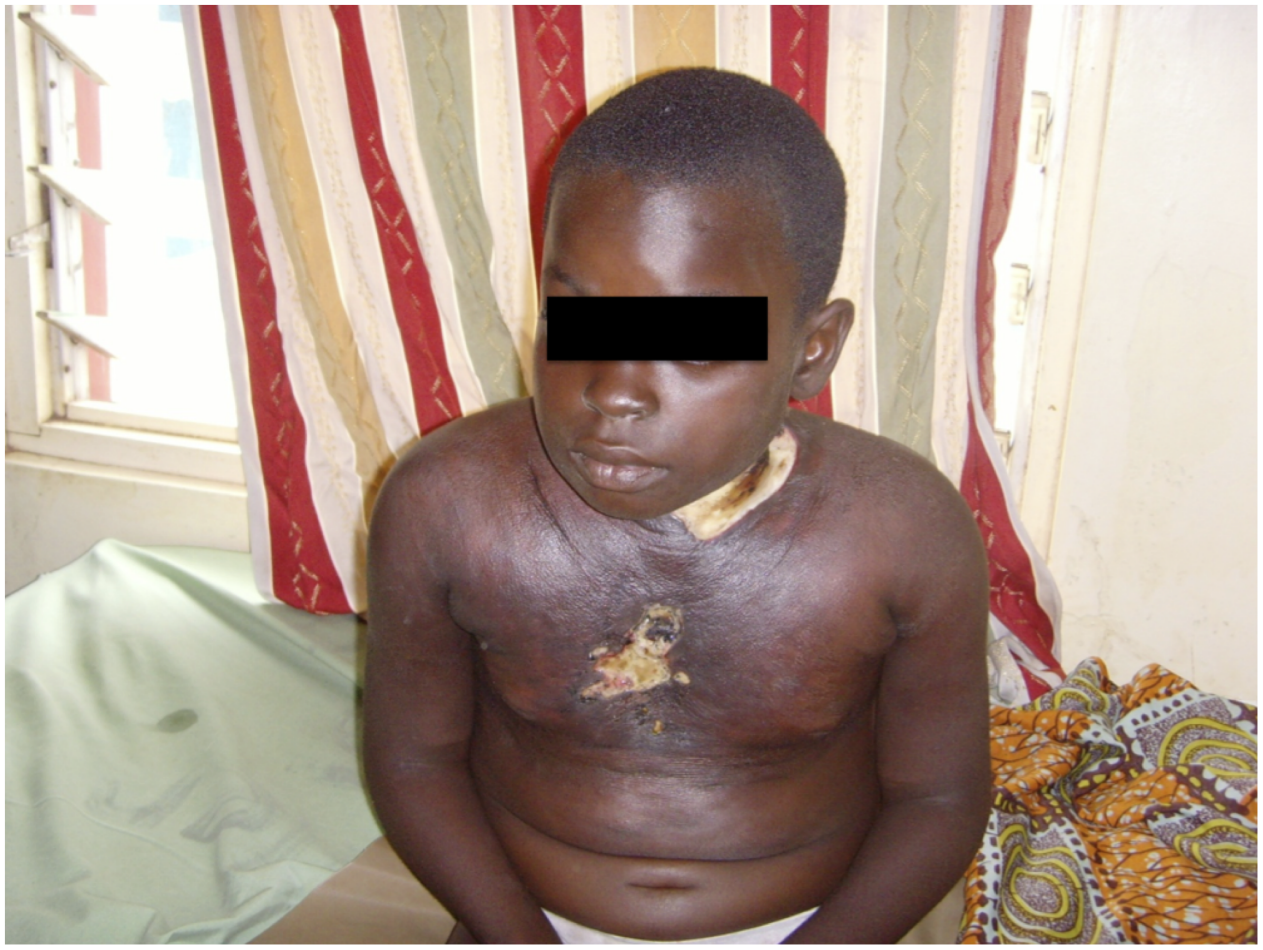
An extensive category III oedematous lesion involving the anterior and posterior chest and neck regions, with two ulcers: one at the anterior triangle of the neck, measuring 6 cm x 8 cm, and another over the midsternal region of the chest, measuring 3 cm x 4 cm.

Patient GO is an 8-year-old student from Drobonso, a Buruli ulcer–endemic community in the Sekyere Afram Plains of the Ashanti region of Ghana. She presented with a month's history of a painless nodule on the anterior chest which started increasing in size rapidly over the course of two weeks. After applying herbal preparations on the lesion and observing no improvement in symptoms, she sought medical attention through which a course of oral amoxycillin was prescribed for a week. However, her lesion continued to enlarge with involvement of her anterior and posterior chest walls and ulcerations over the left anterior cervical triangle and midsternal region. Upon the recommendation of a former patient from the same endemic region, she came to the Buruli ulcer clinic at the Agogo Presbyterian Hospital on the 24th of February 2011. On examination, the patient looked fairly stable and was afebrile (36.9°C), not pale, and anicteric with no regional lymphadenopathy. Her chest was clinically clear with a respiratory rate of 14 cycles/min. The most significant finding was an extensive oedematous lesion involving the chest and neck regions with two deep ulcerations (Figure 1). She weighed 31 kg at diagnosis (95th centile in weight for age). Swab samples obtained from the ulcer were positive by PCR for IS2404, acid-alcohol-fast bacilli, and *Mycobacterium ulcerans* culture on Lowenstein-Jensen culture slopes. Patient was admitted, and antibiotic combination therapy comprising daily intramuscular injection of 15 mg/kg of streptomycin and 10 mg/kg of oral rifampin was started together with wound care and nutritional support.

**Figure 2:**
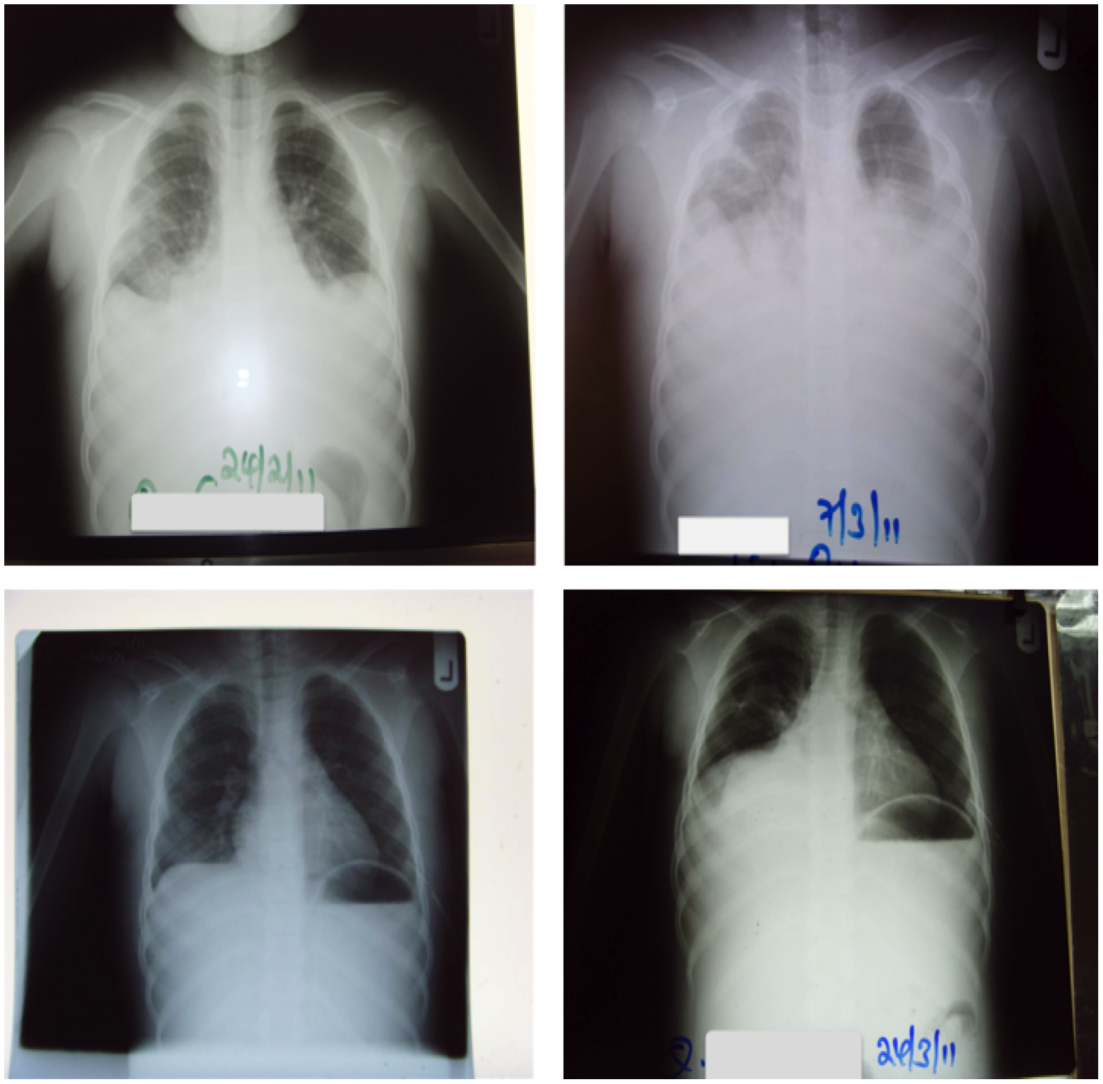
Chest radiographs of patient before and during antibiotic chemotherapy for *M. ulcerans* disease. (A) Chest X-ray taken before antibiotic therapy was started. (B) Chest X-ray taken 2 weeks after initiation of antibiotic therapy, showing bilateral pleural effusions. (C) Week 3 after drainage of pleural effusion. (D) Recurrence of pleural effusion on the right hemithorax at week 4.

Two weeks into treatment, she was noted to have become breathless with the accompaniment of fever and chills but with no associated cough. Clinical examinations revealed the presence of pleural effusions bilaterally and pyrexia of 37.9°C. Full blood counts revealed her white cell count had increased from 10.3 x 10^3^/L on admission to 20.8 x 10^3^/L. An HIV antibody test was negative, and the performance of a chest X-ray confirmed the presence of bilateral pleural effusions (Figure 2). Diagnostic pleural aspirate showed the effusion to be an exudate with an elevated protein concentration of 82.5 g/l, pH = 7.5, and predominantly neutrophils on cytopathology but no isolates on aerobic and anaerobic cultures. PCR on pleural aspirate was positive for *M. ulcerans* but was negative for tuberculosis—a differential diagnosis. An aspirate of the effusion was tested for the presence of mycolactone using UPLC-mass spectrometry, as described elsewhere [1], but the intact mycolactone A/B was not detected. The left pleural effusion was drained with the aid of a chest tube and by needle aspiration of the right pleural effusion. She was put on 30 mg of oral prednisone twice daily for 2 weeks with resolution of the effusion within 2 weeks of its appearance. However, the effusion on the right side recurred (Figure 2D) and was managed successfully by needle thoracocentesis. Upon completion of antimycobacterial antibiotic therapy for 8 weeks, surgery was performed, comprising excision and skin grafting. The lesion healed gradually over 48 weeks but resulted in a hypertrophic scar over the manubrio-sternum. Parental informed consent was obtained.

## Case Discussion

This case report is instructive in illustrating the successes and challenges associated with Buruli ulcer management in rural communities in endemic countries and in highlighting a rare presentation of paradoxical reaction of *M. ulcerans* disease. Combination antibiotic therapy comprising daily oral rifampin and intramuscular streptomycin for 8 weeks has demonstrated excellent efficacy in healing all forms and categories of *M. ulcerans* disease, with surgery sometimes necessary in large category III lesions (lesions >15 cm at its widest diameter) [2]. However, antibiotic therapy for *M. ulcerans* disease may be associated with paradoxical exacerbations or with the appearance of secondary lesions at different sites, often referred to as a paradoxical reaction [3]–[6].

Paradoxical reaction or exacerbation of mycobacterial infections during antibiotic therapy is a well-characterised phenomenon, and in *M. ulcerans* disease a few case reports have been published [3]–[6]. However, to the best of our knowledge, this is the first description of pleural effusion in association with antibiotic-treatment-associated paradoxical reaction in *M. ulcerans* disease. In *M. ulcerans* disease, paradoxical reactions often are characterised by the occurrence of inflammatory pus-filled lesions either close to the primary site of the lesion or sometimes at secondary sites distant from the primary lesions. These lesions either occur during or some weeks after completion of antibiotic therapy. The phenomenon, akin to the immune reconstitution inflammatory syndrome amongst HIV patients on antiretroviral therapy, is thought to be due to an exuberant reconstitution of the local immune response during the demise of *M. ulcerans* bacilli under the influence of antibiotic therapy and mediated by immunostimulators [7] released from dying mycobacteria in a milieu of declining mycolactone concentrations. This is evidenced histologically by massive leucocyte infiltrations of necrotic areas in surgically excised paradoxical lesions in contrast to the acellular to scanty immune cell infiltrates observed in untreated *M. ulcerans–*infected skin lesions.

Although *M. ulcerans* causes a predominantly cutaneous infection, it may occasionally involve deeper structures such as the bone, causing osteomyelitis. Mycolactone has been shown to be cytotoxic to a range of immune and nonimmune cells [8]. It is possible that the manifestation of bilateral pleural effusions in this patient who had an extensive oedematous lesion on the chest wall may signify a local destruction and extension through the chest-wall muscles into the pleural cavities. Against this proposition is the absence of clinically or radiologically demonstrable pneumothoraces. The most probable explanation, however, is that *M. ulcerans* might have been carried into the pleural cavity via the lymphatics to elicit a vigorous inflammatory response once mycolactone concentrations began to decline under the influence of antibiotic therapy. In murine studies, *M. ulcerans* is found to be present in local and regional lymph nodes at some stage during infection [9].

This patient improved on supportive therapy comprising thoracocentesis, a 2-week course of prednisone, completion of the 8-week course of antibiotics, nutritional support, and surgical therapy including debridement and skin grafting, with her lesions healing without recurrence.

**Key Learning Points**

- *M. ulcerans* disease of the chest wall may rarely be associated with exudative pleural effusions.
- *M. ulcerans* cultures of pleural aspirates were negative, which is typical of paradoxical reactions.
- Management of pleural effusions due to paradoxical reactions should include thoracocentesis, steroid therapy, and management of ulcers along conventional lines.
